# Evidence of suppression of onchocerciasis transmission in the Venezuelan Amazonian focus

**DOI:** 10.1186/s13071-016-1313-z

**Published:** 2016-01-27

**Authors:** Carlos Botto, María-Gloria Basañez, Marisela Escalona, Néstor J. Villamizar, Oscar Noya-Alarcón, José Cortez, Sarai Vivas-Martínez, Pablo Coronel, Hortencia Frontado, Jorge Flores, Beatriz Graterol, Oneida Camacho, Yseliam Tovar, Daniel Borges, Alba Lucia Morales, Dalila Ríos, Francisco Guerra, Héctor Margeli, Mario Alberto Rodriguez, Thomas R. Unnasch, María Eugenia Grillet

**Affiliations:** Centro Amazónico de Investigación y Control de Enfermedades Tropicales Servicio Autónomo CAICET, Ministerio del Poder Popular para la Salud, Puerto Ayacucho, Estado Amazonas Venezuela; Instituto de Medicina Tropical, Facultad de Medicina, Universidad Central de Venezuela, Caracas, Venezuela; London Centre for Neglected Tropical Disease Research, Department of Infectious Disease Epidemiology, Faculty of Medicine (St Mary’s campus), Imperial College London, London, UK; Cátedra de Salud Pública. Facultad de Medicina (Escuela Luis Razetti), Universidad Central de Venezuela, Caracas, Venezuela; Instituto de Altos Estudios “Dr. Arnoldo Gabaldón”, Ministerio del Poder Popular para la Salud, Maracay, Estado Aragua Venezuela; Instituto Geográfico de Venezuela “Simón Bolívar”, Caracas, Venezuela; Instituto Nacional de Investigaciones Agrícolas, Puerto Ayacucho, Estado Amazonas Venezuela; Onchocerciasis Elimination Program for the Americas (OEPA), Guatemala City, Guatemala; Centro de Biotecnología Genómica, Instituto Politécnico Nacional, Reynosa, Mexico; Department of Global Health, University of South Florida, Tampa, FL USA; Laboratorio de Biología de Vectores y Parásitos, Instituto de Zoología y Ecología Tropical, Facultad de Ciencias, Universidad Central de Venezuela, Apartado Postal 47072, Caracas, 1041-A Venezuela

**Keywords:** Onchocerciasis, Transmission, Suppression, *Simulium guianense* sensu lato, Ivermectin, Yanomami, Amazonas, Venezuela

## Abstract

**Background:**

The World Health Organization (WHO) has set goals for onchocerciasis elimination in Latin America by 2015. Most of the six previously endemic countries are attaining this goal by implementing twice a year (and in some foci, quarterly) mass ivermectin (Mectizan®) distribution. Elimination of transmission has been verified in Colombia, Ecuador and Mexico. Challenges remain in the Amazonian focus straddling Venezuela and Brazil, where the disease affects the hard-to-reach Yanomami indigenous population. We provide evidence of suppression of *Onchocerca volvulus* transmission by *Simulium guianense* s.l. in 16 previously hyperendemic Yanomami communities in southern Venezuela after 15 years of 6-monthly and 5 years of 3-monthly mass ivermectin treatment.

**Methods:**

Baseline and monitoring and evaluation parasitological, ophthalmological, entomological and serological surveys were conducted in selected sentinel and extra-sentinel communities of the focus throughout the implementation of the programme.

**Results:**

From 2010 to 2012–2015, clinico-parasitological surveys indicate a substantial decrease in skin microfilarial prevalence and intensity of infection; accompanied by no evidence (or very low prevalence and intensity) of ocular microfilariae in the examined population. Of a total of 51,341 *S. guianense* flies tested by PCR none had L3 infection (heads only). Prevalence of infective flies and seasonal transmission potentials in 2012–2013 were, respectively, under 1 % and 20 L3/person/transmission season. Serology in children aged 1–10 years demonstrated that although 26 out of 396 (7 %) individuals still had Ov-16 antibodies, only 4/218 (2 %) seropositives were aged 1–5 years.

**Conclusions:**

We report evidence of recent transmission and morbidity suppression in some communities of the focus representing 75 % of the Yanomami population and 70 % of all known communities. We conclude that onchocerciasis transmission could be feasibly interrupted in the Venezuelan Amazonian focus.

**Electronic supplementary material:**

The online version of this article (doi:10.1186/s13071-016-1313-z) contains supplementary material, which is available to authorized users.

## Background

Onchocerciasis is a chronic and cumulative skin and ocular disease caused by infection with the filarial nematode *Onchocerca volvulus* Leuckart and transmitted among humans through the bites of blackfly species of the genus *Simulium* Latreille. The embryonic stages of the parasite (microfilariae, mf) migrate through the skin and cause severe itching, skin disease, and ocular lesions, with the severity of the clinical manifestations depending on the length of exposure to blackfly bites and the density of mf in the skin [1, 2]. Visual loss and blindness can result from exposure to heavy parasite loads in the human host over time [2]. Since blackflies breed in fast flowing rivers, the disease is also known as ‘river blindness’, although in the Americas it is called Robles’ Disease after Rodolfo Robles, who described it one hundred years ago in Guatemala [3].

In the Americas, the infection was formerly prevalent in 13 endemic foci distributed in 6 countries (Brazil, Colombia, Ecuador, Guatemala, Mexico and Venezuela), where 565,232 persons were considered at risk of infection [4]. In Venezuela, there were three onchocerciasis foci (north-eastern, north-central, and southern), of which, the latter remains as the only persistent focus of the infection in the country [4, 5]. The southern focus comprises endemic areas in the rainforest of the Upper Orinoco, Upper Siapa and Upper Caura River basins (in the Venezuelan Guayana), affecting the Yanomami indigenous group and extending beyond the border with Brazil to join the Yanomami Brazilian area to form the onchocerciasis Amazonian focus [6]. This is the largest focus by area in Latin America, affecting 26,715 people [4], of which 13,231 (49.5 %) are in the Venezuelan part of the focus. The southern focus shows an epidemiological spatial gradient, including areas of high transmission intensity with substantial levels of cutaneous and ocular morbidity observed prior to the start of the elimination programme. In the hyperendemic communities of the focus, skin disease was highly prevalent, with 24 % of the population affected by lichenified onchodermatitis and 10 % suffering from skin atrophy [7]. The pre-treatment prevalence of onchocercal nodules (onchocercomata), especially on the head, was 29 %, reaching 51 % in some communities (e.g., in Orinoquito). Presence of lymphatic lesions―including hanging groin―previously described in Africa was also reported [8]. Similarly, ocular pathology—up to 50 % prevalence of punctate keratitis, mainly due to the presence of mf in the cornea (MFC) and up to 75 % prevalence of mf in the anterior chamber of the eye (MFAC)—was a major clinical manifestation attributable to onchocerciasis. In some hyperendemic communities of the Parima area, the prevalence of any onchocerciasis-associated ocular lesions was greater than 50 %, reaching up to 70 % in those individuals aged ≥40 years. The prevalence of irreversible ocular lesions such as sclerosing keratitis (cumulative inflammatory lesions in the cornea that do not regress but cause progression to eye damage and irrecoverable loss of vision) reached up to 17 % in the Orinoquito area. Bilateral blindness due to onchocerciasis was observed in 0.45 % of the general population [8].

*Simulium guianense* sensu lato (s.l.) Wise, *S. incrustatum* Lutz, and *S. oyapockense* s.l. Floch and Abonnenc are the main vectors in the Southern Focus of Venezuela, with the former species being the most competent for *O. volvulus* and the predominant human-biting blackfly in most of the hyperendemic areas of the focus [9, 10]. *Simulium incrustatum*, with a lower vector competence than *S. guianense* s.l., contributes to the transmission of onchocerciasis in mesoendemic and some hyperendemic areas, whereas *S. oyapockense* is the main vector in hypoendemic communities with low intensity of transmission [10–12].

The strategy adopted by the Onchocerciasis Elimination Program for the Americas (OEPA) since its commencement in 1993 has included elimination of new (ocular) morbidity caused by *O. volvulus*, and interruption of transmission by 6-monthly mass administration of ivermectin (Mectizan®, donated by Merck & Co Inc), delivered by mobile teams with a therapeutic coverage ≥85 % of eligible population in all the endemic communities of the region, including hypoendemic areas [13, 14]. (Given that, on average, approximately 15 % of the population are commonly not eligible for ivermectin treatment, this translates into a therapeutic coverage ≥70 % of the total population.) Ivermectin kills the mf and temporarily inhibits their release by gravid adult female worms [15], as well as killing adult worms after several years of mass treatment given at 6-monthly intervals [16, 17]. More recently, 3-monthly treatments have been introduced in some communities in Mexico and Venezuela [6, 18], given the results of clinical trials conducted in Guatemala [19] and Africa [20]. The OEPA strategy has led to the elimination of incident cases of ocular disease and the interruption of transmission in 11 of the formerly 13 endemic foci [4]; the two remaining foci being the Venezuelan and the Brazilian parts of the Amazonian focus.

The present work reports on the progress towards onchocerciasis elimination in southern Venezuela, according to the protocols proposed by the World Health Organization (WHO), which include in-depth parasitological, entomological and serological surveys, as well as guidance on operational thresholds [21]. Specifically, we report evidence of recent suppression (as defined in [21]) of *O. volvulus* transmission by *S. guianense* s.l. in 16 sentinel and extra-sentinel (previously hyperendemic) communities localized in different geographical areas of the southern Venezuelan focus after 15 years of 6-monthly and 5 years of 3-monthly mass drug administration (MDA) of ivermectin.

## Methods

### Ethics approval and consent to participate

The parasitological, clinical, entomological and serological studies received ethical clearance from the Ethics Review Committee of CAICET (as part of the Ministerio del Poder Popular para la Salud–Venezuelan Ministry of Health). All the participants signed an informed consent form before undergoing any examination, testing, or agreeing to help as human attractants for entomological collections. Additionally, there was active participation of Yanomami volunteers (and their organization “Horonami”) in the identification of new communities and distribution of ivermectin across the focus.

### Study area and study population

**Fig. 1:**
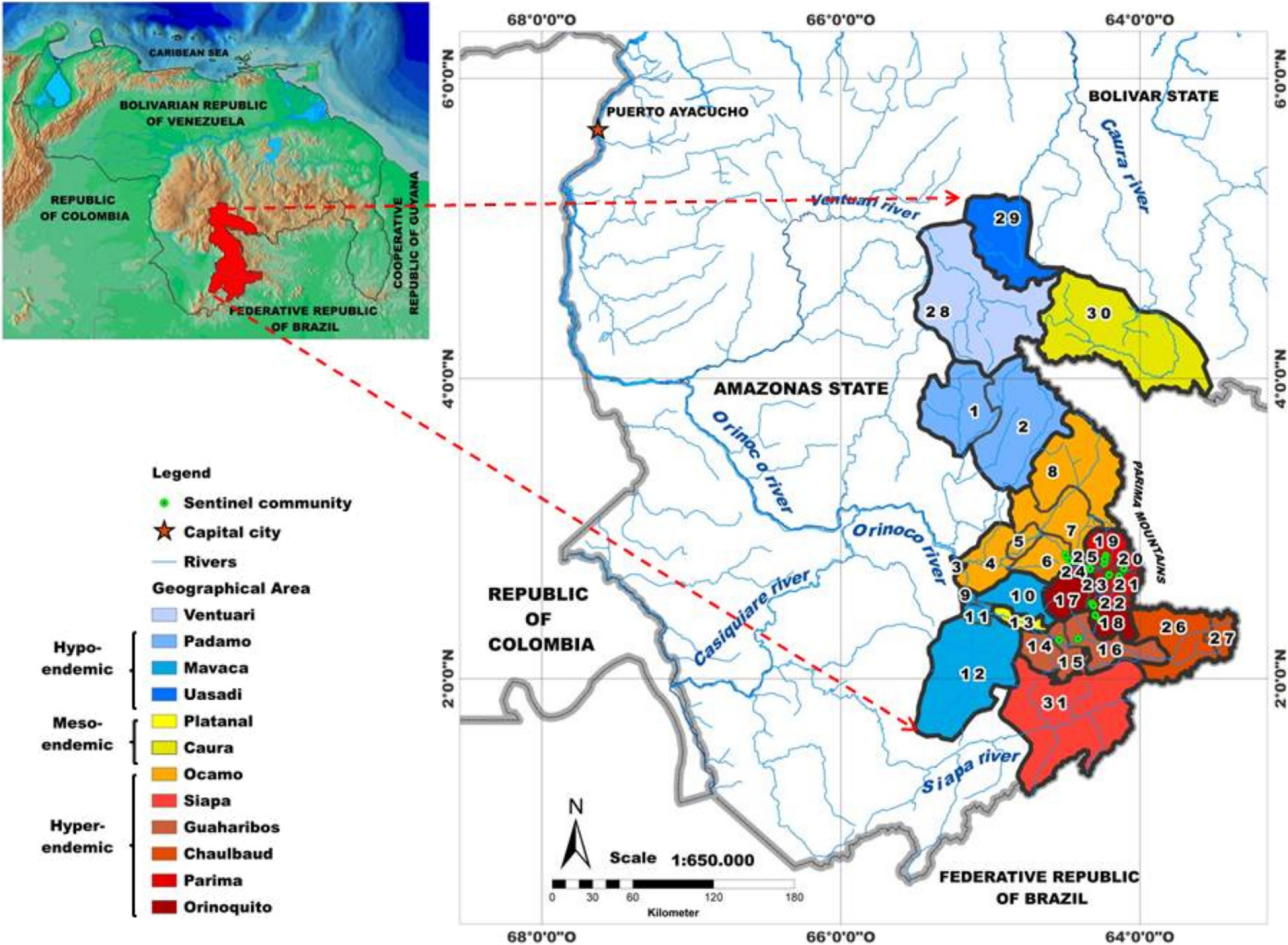
Venezuelan part of the Amazonian onchocerciasis focus. The legend lists the 12 geographical areas of the focus coloured by baseline endemicity of *Onchocerca volvulus* infection, from lowest (light blue) in Ventuari to highest (dark red) in Orinoquito. The numbers indicate the 31 geographical sub-areas described in Table 1

Onchocerciasis transmission in southern Venezuela occurs in the lowlands (0–500 m above sea level, asl) and uplands (500–1200 m asl) of the Upper Orinoco, Upper Siapa and Upper Caura River basins (in the Amazonas and Bolivar States), which are part of the ancient Guayana Shield of northern South America, the oldest (3600 million years) region of the world (Fig. 1). Additional file 1 describes in detail the geographical and environmental characteristics of the focus pertinent to the transmission of onchocerciasis (see *Text S1. Geographical and environmental characteristics of the Venezuelan part of the Amazonian onchocerciasis focus*). The main onchocerciasis transmission seasons (regardless of the *Simulium* vector species present), occur during the dry to rainy (February–April) and rainy to dry (September–November) transitions [11]. The Yanomami indigenous group is the human population afflicted by onchocerciasis in the Amazonian focus, with more than 25,000 inhabitants and four distinct linguistic subgroups (Yanomami, Yanomam, Yanam, and Sanemá), of which the Yanomami is the most affected in Venezuela (Amazonas State), followed by the Sanemá (Bolivar State).

The Venezuelan part of the focus encompasses 12 geographical areas: Padamo; Ocamo; Mavaca; Platanal; Guaharibos; Orinoquito; Parima; Chalbaud; Ventuari; Uasadi; Caura, and Siapa. Within these 12 main areas, 31 geographical sub-areas have been described (Fig. 1 and Table 1). Further details of the endemic communities, mapping and geographical information system can be found in Additional file 1 (see *Text S2. Endemic communities, mapping and geographical information system*).

**Table 1:** Onchocerciasis endemic communities by geographical area and sub-area, population at risk and population eligible for ivermectin treatment in the Amazonian focus of southern Venezuela

Geographical area	Geographical sub-area^a^	No of communities per endemicity level^b^			Population at risk	Eligible population (%)
		Hyperendemic	Mesoendemic	Hypoendemic		
Padamo	1. Upper Padamo	0	0	6	617	529 (85.7)
	2. Upper Cuntinamo	0	3	1	225	188 (83.6)
Ocamo	3. Ocamo–Orinoco	0	0	4	245	223 (91.0)
	4. Lower Ocamo	0	2	5	279	249 (89.3)
	5. Middle Ocamo	0	5	1	508	481 (94.7)
	6. Jénita–Putaco	4	0	0	218	192 (88.1)
	7. Upper Ocamo–Shitari	17	2	1	755	660 (87.4)
	8. Upper Ocamo–Parima	16	4	0	589	497 (84.4)
Mavaca	9. Mavaca–Orinoco	0	0	12	927	804 (86.7)
	10. Manaviche	0	1	1	140	129 (92.1)
	11. Mavaca	0	0	3	216	193 (89.4)
	12. Mavaquita	0	0	11	944	841 (89.1)
Platanal	13. Platanal	3	70	10	569	506 (88.9)
Guaharibos	14. Unturán	5	0	0	338	307 (90.8)
	15. Guaharibos	2	0	0	139	123 (88.5)
	16. Peñascal	3	0	0	255	216 (84.7)
Orinoquito	17. Mayo	8	0	0	477	407 (85.3)
	18. Orinoquito	14	0	0	795	688 (86.5)
Parima	19. Parima B	25	0	0	1045	843 (80.7)
	20. Parima C	9	0	0	609	505 (82.9)
	21. Parima A	19	0	0	917	789 (86.0)
	22. Porewë	7	0	0	247	218 (88.3)
	23. Pasumopë	5	0	0	296	260 (87.8)
	24. Shamatari	4	0	0	289	250 (86.5)
	25. Posheno	3	0	0	146	121 (82.9)
Chalbaud	26. Hashimú	7	0	0	356	321 (90.2)
	27. Chalbaud	10	0	0	300	258 (86.0)
Ventuari	28. Upper Ventuari	0	0	1	136	118 (86.8)
Uasadi	29. Uasadi	0	0	2	176	148 (84.1)
Caura	30. Upper Caura	0	1	0	72	68 (94.4)
Siapa	31. Upper Siapa	6	0	0	406	324 (79.8)
Total (%)		167 (69.3)	25 (10.4)	49 (20.3)	13,231	11,456 (86.6)

^a^ The numbering of the geographical sub-areas corresponds to that indicated in the map of Fig. 1

^b^ Endemicity levels defined as: hyperendemic, microfilarial prevalence ≥60 %; mesoendemic, mf prevalence = 20–59 %; hypoendemic, mf prevalence <20 % [24]

The (mostly semi-nomadic) Yanomami at-risk population has been estimated as 13,231 people, living in 241 ‘shaponos’ scattered deep in the forest, and practicing shifting cultivation, hunting, fishing and gathering of forest products [6]. Their scanty clothing leads to an almost continuous exposure to biting blackflies. Further details on how the anthropological features of the Yanomami influence their exposure to onchocerciasis have been presented elsewhere [6, 22, 23].

### Baseline endemicity and survey communities for monitoring and evaluation

According to OEPA guidelines [24], those communities with a microfilarial prevalence lower than 20 % are classified as hypoendemic; those with prevalence between 20 and 59 % as mesoendemic, and those communities with a prevalence of 60 % or greater as hyperendemic. Table 1 presents the number of communities thus classified and the population at risk and eligible for ivermectin treatment in the 12 geographical areas and 31 geographical sub-areas of the Venezuelan part of the Amazonian focus. The population at risk, 13,231 people as of 2015, was calculated based upon regularly updated demographic censuses conducted by the mobile teams that distribute ivermectin treatment. The population eligible for mass administration of ivermectin, 11,456 (86.6 %) people, were those aged five years or older, excluding those weighing less than 15 Kg (or measuring less than 90 cm in height), pregnant women and those breastfeeding a child younger than one week old (representing 13.4 % of the population).

The pre-treatment levels of endemicity across all geographical areas were very heterogeneous (Table 1), with some areas including communities that were all hyperendemic (e.g., Orinoquito, Parima, Chalbaud), and others where hypoendemic communities prevailed (e.g., Padamo, Mavaca) [25]. In other areas (Ocamo), a gradual increase with increasing altitude in the proportion of hyperendemic communities has been observed [6, 7, 22], with hypoendemic communities in the low reaches of the Ocamo river (Lower Ocamo, 5 communities), mesoendemic communities in the middle reaches of the Ocamo river (Middle Ocamo, 5 communities) and hyperendemic communities in the upper reaches of the Ocamo river (Upper Ocamo–Shitari, 17 communities and Upper Ocamo–Parima, 16 communities), as summarized in Table 1. Overall, of the 241 endemic communities, 167 (69.3 %) were hyperendemic and mostly localized in the Ocamo, Siapa, Guaharibos, Chalbaud, Parima, and Orinoquito areas (Fig. 1), where the predominant anthropophagic blackfly species is *S. guianense* s.l. in most of the localities [6, 22].

The criteria for selection of sentinel and extra-sentinel communities for regular monitoring and evaluation activities, consisting of in-depth epidemiological evaluations included: a) hyperendemic status, b) relative ease of accessibility by the mobile teams that conduct such evaluations, c) existence of historical, baseline epidemiological data prior to wide-spread ivermectin distribution; d) illustrative of the simuliid species composition of the focus. Accordingly, eight communities were selected as sentinel communities, namely (omitting the suffix ‘theri’ that designates the place name for a Yanomami village, for simplicity): (1) Hasupiwei (altitude: 200 m asl; 73 inhabitants) in Guaharibos–Unturán; (2) Awei (162 m asl; 30 inhabitants) and (3) Pashopëka (240 m asl; 89 inhabitants) in Upper Ocamo–Shitari; (4) Koyowë (= Coyowë) (250 m asl; 129 inhabitants), (5) Waharafitha—previously Fubalema—(260 m asl; 97 inhabitants) and (6) Matoa (360 m asl; 48 inhabitants) in Orinoquito; and (7) Kanoshewë (819 m asl; 66 inhabitants) and (8) Niayopë—previously Niyayowë—(950 m asl; 86 inhabitants) in Parima–Shamatari. Since the size of Yanomami communities is generally small, to increase sample size an additional 8 villages were selected as extra-sentinel communities. These were: (9) Yaurawë (198 m asl; 115 inhabitants) in Guaharibos–Unturán; (10) Masiriki (990 m; 44 inhabitants) and (11) Toumawei (1037 m asl; 32 inhabitants) in Parima (A); (12) Arokofita (871 m asl; 67 inhabitants) and (13) Okiamo (927 m asl; 58 inhabitants) in Parima (B); (14) Warapawë (1007 m asl; 110 inhabitants) in Parima (C); and (15) Kakarama (669 m asl; 57 inhabitants) and (16) Pokoshiprare (721 m asl; 90 inhabitants) in the Parima–Shamatari sub-area within the Parima area, the latter two communities derived from Yoreshiana A and Yoreshiana B (see [26]).

### History of mass ivermectin treatment in the Amazonian focus

**Fig. 2:**
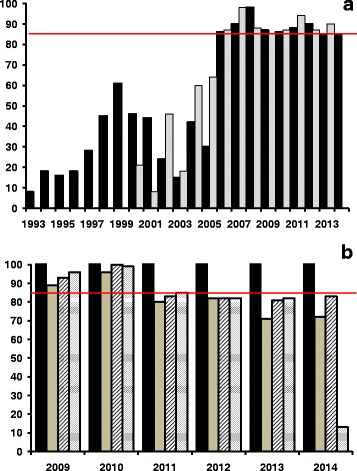
Temporal trends in therapeutic coverage (%) of ivermectin treatment for the eligible Yanomami population in the Venezuelan part of the Amazonian focus. From 1993 through 1999, treatment was distributed annually by mobile teams. In 2000 treatment frequency was increased to twice per year and in 2009 to four times per year. **a** Coverage of annual and twice per year treatment. **b** Coverage of three-monthly treatment since 2009 (black, grey, hatched and dotted bars indicate, respectively, the therapeutic coverage in the first, second, third and fourth quarters of the year). The red horizontal line at 85 % in both **(a)** and **(b)** indicates the minimum coverage of eligibles that needs to be reached and sustained to interrupt transmission according to OEPA’s strategy

**Fig. 3:**
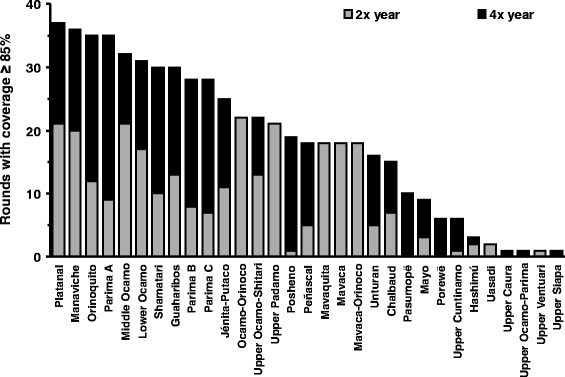
Ivermectin rounds by geographical sub-area in the Venezuelan part of the Amazonian focus. The number of treatment rounds achieving ≥85 % coverage for twice per year and quarterly treatment frequency by decreasing order for the 31 geographical sub-areas of the focus. The grey and black sections of the bars indicate, respectively, the number of rounds that achieved the desired ≥85 % coverage at twice per year and quarterly frequency (Table S1 of Additional file 1)

In the Amazonian focus, annual ivermectin distribution commenced in 1993 only in a few communities, and with a low mean therapeutic coverage (of less than 60 % up to 2000; Fig. 2a). This period is henceforth referred to as ‘pre-ivermectin MDA’, given the low geographical and therapeutic coverage that had been achieved. From 2000 onwards (period henceforth referred to as ‘during ivermectin MDA’), the onchocerciasis elimination programme in Venezuela was drastically re-organised under OEPA’s strategic plan and started 6-monthly ivermectin treatment, with steadily increasing coverage. The 85 % coverage goal (for each treatment round) was reached in 2006 throughout the focus and it has been sustained since then (Fig. 2a). Treatment frequency was further increased from twice to four times per year in 45 communities during 2009 and currently, this quarterly treatment regimen has been extended to 192 out of 241 (80 %) of the endemic communities in the focus, albeit with a slight decreasing trend in coverage for the second to the fourth quarterly rounds (Fig. 2b). This treatment approach was adopted to accelerate interruption of transmission and to accelerate the death of adult worms, especially in areas with very high vector biting density, in communities whose mf prevalence and intensity seemed to have reached a new (lower than baseline) pseudo-equilibrium, or in communities that had been recently identified and incorporated into the programme at later stages. The number of treatment rounds per geographical sub-area that attained a coverage ≥85 % during 1995–2015 was calculated dividing the total number of treatments per sub-area in each round by the eligible population for this period (Fig. 3). Although the quarterly treatment regimen is more difficult to sustain at a consistently high 85 % coverage for each round, particularly for the most remote communities and during some times of the year, the greater frequency of visits to each community has meant that at least two complete treatment rounds with a coverage ≥85 % are received annually by each at-risk community. Table S1 of Additional file 1 provides details, for the 31 geographical sub-areas of the focus, of the number of twice-yearly and quarterly ivermectin rounds achieving ≥85 % therapeutic coverage. (The criteria for transmission suppression as reported in this article are described in Additional file 1: Text S3. World Health Organization (WHO) criteria for onchocerciasis elimination.)

### Parasitological and ophthalmological surveys

Parasitological surveys were carried out in the sentinel and extra-sentinel communities of the focus at baseline (1981), pre-ivermectin MDA (1995–1998, 2000, the latter just before the twice per year treatment), and during ivermectin (twice a year and subsequently quarterly) MDA (2001, 2008, 2013, 2015). The skin snip method was used to determine the prevalence and intensity of *O. volvulus* mf. Two skin biopsies from the (right and left) iliac crests were taken from each examined individual with a 2-mm Holth corneoscleral punch, and incubated for 24 h in buffered saline solution; the emerging mf were counted under an inverted microscope and snips were weighed to express results as mf/mg [8, 26]. When it was not possible to weigh the snips in the field, an average weight of 1.62 mg was used, based on data collected in [22, 26].

Ophthalmological evaluations were carried out during similar periods (1981 for baseline; 1997–1998 and 2000 for pre-ivermectin MDA, and 2001, 2008, 2015, during ivermectin MDA). The prevalence of microfilariae in cornea (MFC) and/or in the anterior chamber (MFAC) was determined by an ophthalmologist experienced in conducting onchocerciasis ocular evaluations for OEPA (HM). Ocular examinations were conducted with a slit-lamp in a darkened area after patients were asked to sit with their head between their legs for 5 min to allow any mf present in the anterior chamber to settle in a visible position in order to determine MFAC [24, 27]. The criterion of [27] was followed of taking into account only non-inflammatory keratitis lesions, with evidence of the presence of live or dead mf in the cornea (punctate keratitis stages A and B), as an indicator of MFC. (According to [27], inflammatory punctate keratitis (stages C, D, and E) is neither specific nor a reliable indicator of onchocerciasis-associated ocular disease.) Baseline and pre-ivermectin MDA data were obtained on i) skin mf prevalence and ii) skin mf intensity as both arithmetic (AM) and Williams (WM) mean numbers of mf/mg (in those aged 5 years and above), iii) community microfilarial load or CMFL (geometric mean number of mf per skin snip (mf/ss) in those aged 20 years and above [28]), iv) MFC prevalence, and v) MFAC prevalence. Additional file 1 describes the calculation of the parasitological indices (mf prevalence, AM, WM, CMFL, MFC and MFAC) (see *Text S4. Calculation of parasitological indices*).

### Entomological evaluations and transmission indices

#### Baseline and pre-ivermectin MDA

Detailed entomological evaluations were carried out between 1982 and 2000 in two sentinel communities (Koyowë (=Coyowë) and Niayopë (=Niyayowë) from the hyperendemic geographical areas of Orinoquito and Parima, respectively. In these communities, the proportion of flies with *O. volvulus* L3 larvae and the mean number of L3 larvae per fly were evaluated using classical dissection methods, examining the abdomens, thoraces, and heads of flies [10]. The baseline transmission indices for *S. guianense* s.l. were calculated according to [29].

#### During ivermectin MDA

In a selection of sentinel (Hasupiwei, Pashopëka, Koyowë) and extra-sentinel (Arokofita) communities, and during several consecutive collection days (e.g., three to five days or until twelve days in some cases), host-seeking *S. guianense* s.l. females were collected throughout the high *O. volvulus* transmission seasons (January–March/February–April or September–November) by using collecting protocols adjusted to the local transmission conditions previously established in the area and known periods of highest biting activity by parous flies [11, 30]. All the simuliid females that landed on two human attractants selected from each community, working simultaneously but positioned at a distance of at least 50 m apart, were caught with manual aspirators by a team of two collectors during the first 50 min of each hour, beginning at 09:00 h and ending at 17:50 h, with one break of two hours at midday (12:00–14:00 h), due to a lull in biting density by parous flies during such period [30], for a total of 7 h of collection each day. Flies were collected before procuring a blood meal. Attractants received ivermectin one week prior to participating in fly collection in order to minimise the possibility of the flies becoming infected with ingested mf (if not caught soon enough after landing on the attractants; which could give positive results in the PCR analyses described below). Whenever possible, the collection teams in each community were the same throughout the surveys to minimize variations resulting from individual differences in catching ability. In the field, all hourly-caught flies were anesthetized with chloroform vapour, identified to species, and counted by community, date, day and hour of the day. The number of collection days depended on the biting density in each community in order to reach a number of at least 6000 flies as described below.

Polymerase chain reaction (PCR) using *O. volvulus*-specific DNA probes has been generally applied to examine pools of flies in the OEPA region [21]. Therefore, for each community, flies were combined into pools containing 200 flies per pool (smaller pools containing remaining flies were also analysed), and the heads and bodies were tested separately for *O. volvulus* using a species-specific PCR assay [31] (conducted by MAR, TRU). Details of protocols for genomic DNA purification and parasite detection have been published elsewhere [32]. To maximize the efficiency of the screening process, insect body pools were analyzed first; if any of those pools were positive, all of the head pools were then analyzed, providing an estimate of the infectivity rate (the prevalence of flies carrying only L3 infective larvae in the head). The Pool Screen® software (Version 2.0; University of Alabama, Birmingham, AL) was used to estimate the proportion of positive head pools in the PCR assay and the associated 95 % confidence intervals (95 % CIs) [33]. This software employs a Bayesian method to calculate the probability of infection of an individual blackfly from the number of positive pools and the size of the pools is used to calculate the infectivity rate in the community. Since parity status (proportion of flies that have already laid a batch of eggs) is a very laborious parameter to determine routinely in the field, OEPA’s entomological criterion for cessation of treatment and commencement of surveillance has been simplified to <1 infective fly per 2000 (0.05 %) flies tested (i.e., assuming that 50 % of flies are parous). To reach this operational threshold, it has been determined that the minimum sample size required to have enough power to detect a statistically significant prevalence of infective flies lower than 0.05 % (i.e. not included in the 95 % CI), given that no infective fly may be found, is at least 6000 flies per community [21, 34]. Additional file 1 provides details of the calculation of the transmission indices (hourly biting rate (*HBR*), seasonal biting rate (*SBR*), seasonal transmission potential (*STP*) and annual transmission potential (*ATP*) (see *Text S5. Calculation of transmission indices*).

### Serological evaluations

Serological evaluations were conducted only during the ivermectin MDA period with the aim of measuring the prevalence in samples of children of IgG4 antibodies to Ov-16—a recombinant *O. volvulus* antigen able to signal prepatent infections*—*[35, 36]. ELISA Ov-16 testing is currently being used for serosurveys of children in areas where transmission is deemed to have been interrupted in the Americas [33, 37, 38]. The serological protocol was as follows [36]. Sterile procedures were used to prick the fingers of all participants and four to six drops of blood (80–120 μL) were absorbed onto Whatman No 2 filter paper. The filter paper blood samples were dried, separated by sheets of paper, and then bundled and stored in sealed plastic bags in a cooler until they were returned to the laboratory where they were stored at −20 °C. Two 6-mm punches of blood-saturated filter paper were placed in a phosphate-buffered saline-Tween 0.05 % and bovine serum albumin 5 % buffer and eluted overnight at 4 °C. The elution was then run in duplicate in a standard ELISA to detect IgG4 antibodies against the Ov-16 recombinant antigen. A 5-year cumulative incidence rate of <1 new case per 1000 susceptible children (0.1 %) is the level acceptable in the OEPA region provided that the appropriate population size is available [21]. Here, and following [34], the prevalence of Ov-16 antibodies is taken as equivalent to this cumulative incidence rate. Consequently, to calculate a prevalence statistically significantly lower than 0.1 % (i.e. with a 95 % CI not including this value), and assuming no positives, a sample size of at least 3000 children <10 years of age is required. In the Amazonian focus, given the small community sizes, it is difficult to find this number of children. Consequently, we aimed to show a prevalence of Ov-16 < 1 %, requiring a minimum sample size of 300 children. We tested 396 children aged <10 years throughout the 16 endemic (sentinel and extra-sentinel) communities included in this study during 2013.

## Results

### Parasitology and ophthalmology

#### Baseline and pre-ivermectin MDA

Pre-treatment data (Table 2) showed high prevalence of microfilarial infection by skin biopsy in sentinel and extra-sentinel communities, ranging from 56 % in Pokoshiprare to 100 % in Waharafitha and Toumawei (in the last two only individuals aged ≥10 years were examined). In general, the levels of initial endemicity varied from hyperendemic to very highly hyperendemic or holoendemic, with 11 out of the 16 communities (69 %) having mf prevalence ≥80 %. The highest values of infection intensity were recorded in Toumawei (AM = 231.2 mf/mg; WM = 102.6 mf/mg; CMFL = 104.7 mf/ss). Regarding ocular onchocerciasis, the prevalence of MFC was lowest in Awei (18 %) and highest in Waharafitha (50 %), the latter also being the community with the highest prevalence of MFAC (75 %).

**Table 2:** Prevalence and intensity of *Onchocerca volvulus* microfilariae (mf) in the baseline and pre-ivermectin MDA period (1981– 2000), in sentinel and extra-sentinel communities of the Amazonian focus of southern Venezuela

Geographical sub-area	Community (altitude, masl)	Positive/examined	Prevalence (%) of skin mf (95 % CI)	AM^†^ (mf/mg)	WM^§^ (mf/mg)	CMFL^‡^ (mf/ss)	MFC^¶^ (%)	MFAC^¦^ (%)
Sentinel communities								
Guaharibos	1 Hasupiwei (200)	39/47^b^	83.0 (69.2, 92.4)	50.4	12.7	21.3	–	–
		36/44^c^	81.8 (67.3, 91.8)	48.7	20.3	43.7	–	–
		39^+,d^	–	–	–	–	46.2	43.6
Jénita –Putaco	2 Awei (162)	15/24^b^	62.5 (40.6, 81.2)	61.3	11.4	52.4	–	–
		15/18^d^	83.3 (58.6, 96.4)	60.6	10.8	14.4	17.6	5.9
	3 Pashopëka (240)	29/38^b^	76.3 (59.8, 88.6)	33.8	9.6	19.7	–	–
		43/51^d^	84.3 (71.4, 93.0)	49.9	14.1	17.4	39.2	0
Orinoquito	4 Koyowë (250)	54/64^a^	84.4 (73.1, 92.2)	146.3	25.7	72.6	–	–
		59/72^c^	81.9 (71.1, 90.0)	80.2	18.8	11.0	–	–
		54^+,d^	–	–	–	–	35.2	13.0
	5 Waharafitha (260)	36/36^e^	100 (90.3, 100)	62.1	23.0	57.7	50.0	75.0
	6 Matoa (360)	51/53^e^	96.2 (87.0, 99.5)	84.4	36.2	50.8	17.0	24.4
Parima B	7 Kanoshewë^*^ (819)	34/48^d^	70.8 (55.9, 83.0)	12.5	3.6	4.6	–	–
		54^+,d^					5.6	0
	8 Niayopë^*^ (950)	120/179^a^	67.0 (59.6, 73.9)	44.5	7.6	43.2	–	10.0
		22^+,d^					4.6	0
Extra-sentinel communities								
Peñascal	9 Yaurawë (198)	19/20^d^	95.0 (75.1, 99.1)	133.3	38.7	68.6	29.3	37.9
Parima A	10 Masiriki (990)	19/21^d^	90.5 (69.6, 98.8)	122.8	26.6	31.1	–	–
	11 Toumawei (1037)	19/19^d^	100 (82.4, 100)	231.2	102.6	104.7	–	–
Parima B	12 Arokofita^*^ (871)	22/31^d^	71.0 (52.0, 85.8)	9.3	2.9	7.6	–	–
	13 Okiamo^*^ (927)	13/36^d^	36.1	8.8	0.98	1.1	–	–
		8^+,d^					25.0	0
Parima C	14 Warapawë (1007)	23/24^d^	95.8 (78.9, 99.9)	79.7	20.1	15.6	–	–
Shamatari	15 Kakarama^**^ (669)	39/47^b^	83.0 (69.2, 92.4)	50.4	12.7	33.8	–	–
	16 Pokoshiprare^**^ (721)	19/33^b^	57.6 (39.2, 74.5)	39.6	4.7	30.0	–	–

^†^ *AM* arithmetic mean no. of mf/mg; ^§^ *WM* geometric mean (of Williams) no. of mf/mg; ^‡^ *CMFL* community microfilarial load, the geometric mean no. of mf per skin snip (ss) in those individuals aged ≥20 years; ^¶^ *MFC* prevalence of mf in cornea; ^¦^ *MFAC* prevalence of mf in the anterior chamber of the eye; ^a^1981, ^b^1995, ^c^1997, ^d^1998, ^e^2000; ^+^examined for ocular lesions only, ^*^the community of Niayopë, formerly called Niyayowë and studied in 1981, included Kanoshewë, Arokofita and Okiamo; therefore, although these communities did not exist as separate entities at the time of the baseline study in 1981, their infection levels are assumed to be the same as those of Niyayowë/Niayopë; ^**^ Kakarama and Pokoshiprare originated from Yoreshiana A and B, studied by [26]

#### During ivermectin MDA

**Fig. 4:**
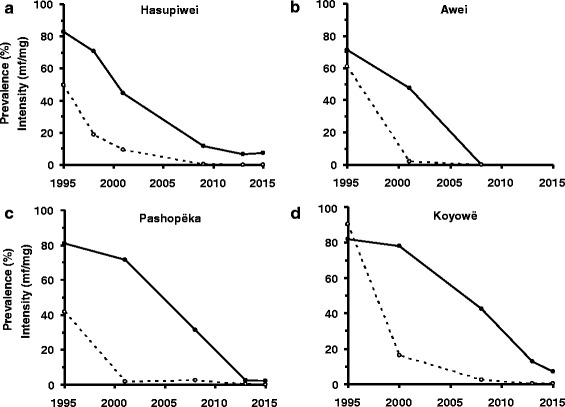
Temporal trends of *Onchocerca volvulus* infection in sentinel communities of the Venezuelan part of the Amazonian focus. For each panel, the baseline values of microfilarial prevalence (%) and intensity (arithmetic mean no. mf/mg) were averaged and plotted for 1995. The solid lines and circles represent infection prevalence, and the dotted lines and open circles represent infection intensity. **(a)** Hasupiwei; **(b)** Awei; **(c)** Pashopëka; **(d)** Koyowë

The results of the parasitological surveys conducted at various time points during ivermectin MDA are shown in Table 3 and, for a selection of communities, graphically in Fig. 4. To avoid parasitological and entomological evaluations being conducted too soon after the last treatment round―which would lead to erroneous conclusions about the effectiveness of the programme―the immediately prior round of treatment was suspended. Therefore, in those communities receiving 6-monthly treatment, epidemiological evaluations were conducted one year after the last treatment round. In those communities receiving 3-monthly treatment, the evaluation surveys were conducted 6 months after the last treatment round.

**Table 3:** Prevalence and intensity of *Onchocerca volvulus* microfilariae during ivermectin MDA (2001–2015), in sentinel and extra-sentinel communities of the Amazonian focus of southern Venezuela

Community	Positive/examined	Prevalence (%) of skin mf (95 % CI)	AM^†^ (mf/mg)	WM^§^ (mf/mg)	CMFL^‡^ (mf/ss)	MFC^¶^ (%)	MFAC^¦^ (%)
Sentinel communities							
1 Hasupiwei	5/43^b^	11.6 (3.9, 25.1)	0.26	0.13	0.11	11.8	0
	4/55^c^	7.3 (2.0, 17.6)	0.13	0.07	0.03	10.0	2.0
2 Awei	11/23^a^	47.8 (26.8, 69.4)	2.06	0.94	3.04	–	–
	0/13^b^	0 (0, 24.7)	0	0	0	0	0
3 Pashopëka	20/28^a^	71.4 (51.3, 86.8)	1.74	0.88	1.04	–	–
	10/32^b^	31.2 (16.1, 50.0)	2.51	0.17	1.60	11.1	7.4
	1/49^c^	2.0 (0.10, 10.9)	0.03	0.02	0.03	4.5	0
4 Koyowë	60/77^a^	77.9 (67.0, 86.6)	16.4	4.7	20.2	36.0	13.0
	24/58^b^	41.4 (28.6, 55.1)	3.4	1.1	1.9	20.4	6.1
	7/98^c^	7.1 (2.9, 14.2)	0.18	0.09	0.17	11.9	0
5 Waharafitha	39/80^b^	48.8 (37.4, 60.2)	3.33	0.91	1.19	7.5	12.5
	5/40^c^	12.5 (4.2, 26.8)	0.22	0.12	0.17	0	0
6 Matoa	14/35^b^	40.0 (23.9, 57.9)	3.66	0.91	1.03	8.3	0
7 Kanoshewë	1/11^b^	9.1 (0.2, 41.3)	0.06	0.05	0	–	–
	0/25^c^	0 (0, 13.7)	0	0	0	–	–
8 Niayopë^*^	4/12^b^	33.3 (9.9, 65.1)	0.49	0.17	0	–	–
	0/38^c^	0 (0, 9.3)	0	0	0		
Extra-sentinel communities							
9 Yaurawë	11/37^b^	29.7 (15.9, 47.0)	0.99	0.43	0.55	20.0	16.0
	16/77^c^	20.8 (12.4, 31.5)	1.75	0.40	0.7	11.1	3.7
10 Masiriki	0/21^c^	0 (0, 16.1)	0	0	0^c^	–	–
11 Toumawei	2/25^c^	8.0 (1.0, 26.0)	0.10	0.05	0.10	–	–
12 Arokofita	0/21^c^	0 (0, 16.1)	0	0	0	–	–
13 Okiamo	0/38^c^	0 (0, 9.3)	0	0	0	–	–
14 Warapawë	3/30^b^	10.0 (2.1, 26.5)	0.04	0.04	–	–	–
	0/31^c^	0 (0, 11.2)	0	0	0		
15 Kakarama^**^	1/41^c^	2.4 (0.1, 12.9)	0.03	0.02	0.05	–	–
16 Pokoshiprare^**^	0/60^c^	0 (0, 6.0)	0	0	0	–	–

^†^ *AM* arithmetic mean no. of mf/mg, ^§^ *WM* geometric mean (of Williams) no. of mf/mg; ^‡^ *CMFL* community microfilarial load, the geometric mean no. of mf per skin snip (ss) in those individuals aged ≥20 years; ^¶^ *MFC* prevalence of mf in cornea; ^¦^ *MFAC* prevalence of mf in the anterior chamber of the eye; ^a^2001, ^b^2008–2009, ^c^2013– 2015; ^*^formerly called Niyayowë; ^**^Kakarama and Pokoshiprare originated from Yoreshiana A and B, studied by [26]

The prevalence of *O. volvulus* mf in most of the communities declined markedly from pre-treatment levels (58 to 100 % reduction). According to the results of the most recent epidemiological evaluation (2008–2009 for Awei and 2013–2015 for the remainder), 8 out of 16 (Awei, Kanoshewë, Niayopë, Masiriki, Arokofita, Okiamo, Warapawë, and Pokoshiprare) had 0 mf in skin (and eyes), and 7 out of the 8 remaining communities had CMFL <1 mf/ss. A striking decline was also observed in the prevalence of MFC (Fig. 4a) and MFAC (Fig. 4b), with the prevalence of MFAC decreasing to zero in 5 communities.

**Fig. 5:**
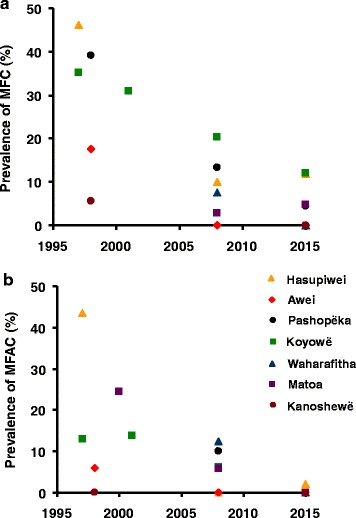
Temporal trends of ocular onchocerciasis prevalence in sentinel communities of the Venezuelan part of the Amazonian focus. **a** Prevalence of microfilariae in the cornea (MFC). **b** Prevalence of microfilariae in the anterior chamber of the eye (MFAC). Orange triangles: Hasupiwei; red diamonds: Awei; black circles: Pashopëka; green squares: Koyowë; blue triangles: Waharafitha; purple squares: Matoa; brown circles: Kanoshewë

By contrast, the communities of Hasupiwei, Pashopëka, Koyowë, Kakarama, Waharafitha, Matoa, Yaurawë, and Toumawei still show mf in skin (and eyes), with prevalence of MFC as high as 12 % (Fig. 5a, d). Of these communities, the latter four had an initial prevalence of microfilaridermia ≥95 %.

### Entomological evaluations

#### Baseline and pre-ivermectin MDA

Table 4 presents data on biting and infectivity rates of *S. guianense* s.l. in two sentinel communities prior to ivermectin MDA. Biting rates were high, particularly in Koyowë, where the monthly biting rate (*MBR*) reached values up to 23,997 bites person^−1^ month^−1^ and the monthly transmission potential (*MTP*) up to 576 L3 person^−1^ month^−1^. Seasonal biting rates (during the higher transmission months of January through March/April plus October through November) were in excess of 100,000 bites per person per transmission season. As these flies were processed by manual dissection, it was possible to determine both the proportion of infective flies and the number of L3 in the flies, with the latter ranging from 0.001 to 0.036 L3/fly (Table 4). In Niayopë (=Niyayowë) the biting rates and transmission indices were lower, with an average *MBR* of 727 bites person^−1^ month^−1^, a seasonal biting rate of roughly 3000 flies per person per transmission season, and a maximum *MTP* of 15 L3 person^−1^ month^−1^. Additional file 1 illustrates the dynamics of biting rates and transmission potentials throughout the year for the baseline entomological studies conducted in Koyowë and Niyayowë (see Figures S1–S3), as well as the relationship between the proportion of infective flies and the mean number of L3 larvae per fly (Additional file 1: Figure S4). Additional file 1: Table S2 provides a comparison of manual dissection and PCR for a *S. guianense* s.l. population sample obtained during May 2000. The corresponding infectivity rates, 0.26 % (95 % CI 0.10–0.53 %) by dissection and 0.38 % (95 % CI 0.19–0.69 %) by PCR are in agreement with the value of 0.37 % for the entire baseline study period (Table 4).

**Table 4:** Biting rate, infectivity rate, mean number of *O. volvulus* L3 per fly, and transmission potentials of *S. guianense* s.l. in the baseline and pre-ivermectin MDA period (1981–2000) in two sentinel communities of the Amazonian focus of southern Venezuela

Community (years)	L3-positive/dissected	*MBR* ^a^ (95 % CI)	*SBR* ^b^ (95 % CI)	Infectivity rate (%) (95 % CI)	No. L3/fly (range)	*MTP* ^c^ (range)	*STP* ^d^	*ATP* ^e^
Koyowë (1982–2000)	108/29,490	20,420 (16,843–23,997)	103,516 (81,142–125,889)	0.37 (0.30–0.44)	0.0079 (0.001–0.036)	179 (14–576)	1406	2020
Niayopë^f^ (1982–1993)	31/4742	727 (417–1036)	2920 (2538–3301)	0.65 (0.44–0.93)	0.0143 (0.001–0.036)	6 (0–15)	40	72

^a^ *MBR*: Monthly biting rate = arithmetic mean number of bites per person per month

^b^ *SBR*: Seasonal biting rate = the number of bites per person per transmission season (January–March plus October–November)

^c^ *MTP*: Monthly transmission potential = number of L3 per person per month = *MBR* × mean number of L3 per fly (located anywhere in the fly’s body)

^d^ *STP*: Seasonal transmission potential = the sum of *MBR* for the months with higher transmission (January–March plus October–November) with L3 larvae located anywhere in the fly’s body

^e^ *ATP:* Annual transmission potential = number of L3 per person per year = the sum of *MBR* values throughout the year with L3 larvae located anywhere in the fly’s body

^f^The community of Niayopë was formerly called Niyayowë

#### During ivermectin MDA

Results of the entomological evaluations carried out from 2006 to 2013 in Hasupiwei, Pashopëka, Koyowë and Arokofita are presented in Table 5. The seasonal biting rates in Koyowë were consistent with those recorded during 1982–2000 (Table 4), suggesting that any decline in transmission is not due to secular environmental and ecological changes affecting vector abundance but likely due to the treatment programme. In 2006, after 12 rounds of ivermectin treatment with ≥85 % of coverage, 7 out of 203 pools of *S. guianense* s.l. heads from Koyowë were PCR positive, leading to a prevalence of infective flies of 1.4 per 2000 tested flies (0.07 %), in contrast with the 7.4 (0.37 %) infective flies per 2000 dissected flies recorded at baseline (Table 4). This represents an 81 % reduction in infectivity. The estimated seasonal transmission potential was 39 L3 (head) per person per transmission season in contrast with 1406 L3 (all) at baseline (a 97 % reduction). Four years later, in 2010, and after 22 rounds if ivermectin MDA (7 annually from 1993 to 1999; 11 biannually from 2000 to 2008, and 4 quarterly during 2009), all 223 body pools representing 10,882 flies of *S. guianense* s.l. tested were PCR negative. This was also the case in 2012–2013, when 13,117 *S. guianense* s.l. flies were tested. However, the upper 95 % CI for the *STP* in 2010 was 25 and for 2012–2013 it was 19 L3 larvae/person/transmission season (accounting fot uncertainty in the estimates). In Hasupiwei, Pashopëka and Arokofita, after 2–3 years of quarterly ivermectin treatment, all 8085, 6464 and 12,793 flies respectively tested in 2012–2013 were PCR negative (with the upper 95 % CI for *STP* of 3–4 L3/person/transmission season).

**Table 5:** Biting rate, infectivity rate, and onchocerciasis transmission potentials of *S. guianense* s.l. during ivermectin MDA (2006–2013) in sentinel and extra-sentinel communities of the Amazonian focus of southern Venezuela

Community (Year)	Flies collected and analysed	*SBR* ^a^ (95 % CI)	Infectivity rate (%)^b^ (95 % CI)	*STP* ^c^ (95 % CI)
Hasupiwei (2012–2013)	8085	15,806 (13,237–18,858)	0 (0–0.025)	0 (0–3.7)
Pashopëka (2012–2013)	6464	13,048 (11,323–15,026)	0 (0–0.03)	0 (0–3.9)
Koyowë (2006)	10,194	56,051 (47,529–66,093)	0.07 (0.025–0.13)	39.2 (15.1–72.1)
Koyowë (2010)	10,882	72,237 (60,839–85,754)	0 (0–0.035)	0 (0–25.3)
Koyowë (2012–2013)	13,117	130,143 (115,704–146,736)	0 (0–0.015)	0 (0–18.9)
Arokofita (2012–2013)	12,793	40,857 (35,308–47,238)	0 (0–0.01)	0 (0–3.1)

^a^ *SBR*: Seasonal biting rate = Geometric mean number of bites per person per transmission season

^b^Calculated as the number of positive fly heads for *O. volvulus* L3 DNA per 2000 flies examined and expressed as a percent

^c^ *STP*: Seasonal transmission potential = the number of L3 (head only) per person per transmission season = *SBR* × infectivity rate (expressed as a proportion) assuming that an infective fly carries on average one infective larva in the cephalic capsule

### Serological evaluation

Table 6 summarises by geographical sub-area the results of the Ov-16 seroprevalence surveys conducted in 2013. Overall, 26 children aged 1–10 years (from 6 communities) were seropositive out of a total of 396 examined (6.6 %; 95 % CI 4.3–9.5 %). Most of the seropositive children (22/26, 85 %) clustered in 5 communities of the Orinoquito sub-area. However, the prevalence for children aged 1–5 years was of 1.8 % (4/218), with only 3 communities (Koyowë, Matoa and Yaurawë) showing specific antibodies to *O. volvulus* Ov-16 for this age group (Table 6).

**Table 6:** Prevalence of IgG4 antibodies to Ov-16 in children aged 1–10 years tested in 2013, by geographical sub-area in the Amazonian focus of southern Venezuela

Geographic sub-area^a^		1–5 years (positive/examined)	Seroprevalence (%) (95 % CI)	6–10 year (positive/examined)	Seroprevalence (%) (95 % CI)	Total (positive/examined)	Seroprevalence (%) (95 % CI)
6	Jénita–Putaco	0/15	0 (0–21.8)	0/14	0 (0–23.2)	0/29	0 (0–11.9)
15	Guaharibos	0/13	0 (0–24.7)	0/8	0 (0–36.9)	0/21	0 (0–16.1)
16	Peñascal	1/23	4.3 (0.1–21.9)	2/15	13.3 (3.7–37.9)	3/38	7.9 (1.7–21.4)
18	Orinoquito	3/62	4.8 (1.7–13.3)	19/54	35.2 (23.8–48.5)	22/116	19.0 (12.9–27.0)
19	Parima B	0/41	0 (0–8.6)	0/32	0 (0–10.9)	0/73	0 (0–4.9)
20	Parima C	0/18	0 (0–18.5)	0/10	0 (0–30.9)	0/28	0 (0–12.3)
21	Parima A	0/9	0 (0–33.6)	0/15	0 (0–21.8)	0/24	0 (0–14.3)
24	Shamatari	0/37	0 (0–9.5)	1/30	3.3 (0.08–17.2)	1/67	1.5 (0.04–8.0)
	Total	4/218	1.8 (0.5–4.6)	22/178	12.4 (8.3–18.0)	26/396	6.6 (4.3– 9.5)

^a^Numbering of sub-areas as in Table 1 and Fig. 1

## Discussion

In this paper we present a compendium of the parasitological, ophthalmological, entomological and serological data obtained in hyperendemic communities of the Venezuelan part of the Amazonian onchocerciasis focus since the original studies conducted in the Parima and Orinoquito areas in 1981 [8]. These studies, and those presented previosuly [7, 22, 25, 26] provided the epidemiological baseline situation prior to the introduction of ivermectin MDA. Both the geographical and therapeutic coverage of annual administration were low when the programme first started in a few communities in 1993 (Koyowë, Kanoshewë, Niayopë). In 2000 a twice per year treatment strategy was adopted, and in 2009, treatment frequency was increased to four times per year (Fig. 2).

Overall, skin microfilarial prevalence and intensity have declined substantially, with reductions in prevalence ranging from 58 % (Matoa) in 2009 to 100 % (Awei, Niayopë, Masiriki, Arokofita, Okiamo, Warapawë and Pokoshiprare) in 2015. By 2015, CMFL and MFAC have become, respectively, negative in 7/16 (44 %) and 5/7 (71 %) of the communities examined for these indicators. Communities of the Orinoquito sub-area (Koyowë, Waharafitha and Matoa) are still positive for *O. volvulus* mf in skin and eyes, likely due to their holoendemic status at baseline and the very high vector biting rates characteristic of the Orinoquito ranforest bioclime (~245,000 bites person^−1^ year^−1^ and 104,000 bites per transmission season in Koyowë, Table 4). This is despite these communities having received 35 rounds of ivermectin with a coverage ≥85 % of eligibles (~75 % of the total population) by 2015 (Fig. 2c). By contrast, communities located in the Parima sub-areas (Niayopë, Masiriki, Toumawei, Arokofita, Okiamo, Warapawë, Kakarama and Pokoshiprare) have experienced greater reductions in mf prevalence (ranging from 92 to 100 %), probably due to the lower vector density of *S. guianense* s.l. in this highland savannah bioclime (8700 bites person^−1^ year^−1^ and 2920 bites per transmission season as recorded in Niayopë, Table 4).

In the OEPA region, absence or near absence of L3 larvae in the head of blackfly vectors (as measured by pool-screen PCR in samples of 6000–10,000 flies), a 99 % reduction in the intensity of transmission (as measured by seasonal transmission potentials), and the absence of detectable *O. volvulus* infection (by parasitological or immunological diagnostics) in children have been the WHO criteria followed to certify focal interruption of parasite transmission [21]. Prior to reaching this epidemiological status, the focus starts to show declining to very low or negative parasitological results in skin, eyes (indicators of reversible morbidity) and flies, suggesting that transmission of the infection has been suppressed by the treatment [21, 34]. Here, we report 81 % reductions in fly infectivity and 97 % reductions in seasonal transmission potentials, with an overall prevalence of 7 % in Ov-16 seroprevalence among children aged up to 10 years and of 2 % among those under 5 years, providing evidence of suppression of *O. volvulus* transmission by the most competent vector of the focus, *S. guianense* s.l., in areas formerly hyperendemic to holoendemic. The dramatic decline in the seroprevalence among children and the lack of evidence of parasite-vector contact suggest that four times per year treatment has successfully suppressed transmission in many of the endemic communities. It will now be necessary to maintain pressure on the parasite population, continuing to suppress transmission, until the existing adult female parasites are either killed or rendered sterile by repeated ivermectin treatments [20].

Ivermectin is expected to have a faster impact in those areas of the Amazonian focus with lower vector competence blackfly species (e.g. *S. oyapockense* s.l. and/or *S. incrustatum* [12, 39], which sustain hypo- to mesoendemic transmission [22], or where vector biting rates are lower and perhaps closest to critical biting rates necessary to maintain endemic transmission (basic reproduction ratio, *R*_0_ ≥ 1 [40]). These threshold biting rates do not only depend on vector competence for *O. volvulus*, but also on the human blood index (HBI, the proportion of vector blood meals of human origin) of the various blackfly species and populations therein. At present, this parameter is unknown for the simuliid species prevailing in the Amazonian focus, but field and theoretical studies on *S. damnosum* s.l. in West Africa indicate that the HBI can be highly variable [41] and possibly host and fly density dependent [42]. Given that the Venezuelan part of the Amazonian focus is sparsely inhabited by human populations, it is likely that the HBI is relatively low and threshold biting rates correspondingly high. Serological data (albeit with low sample sizes reflected in the 95 % CI shown in Table 6) suggest that suppression of transmission may have been more rapidly accomplished in those communities with seasonal biting rates (*SBR*s) of *S. guianense* s.l. lower than 50,000 bites/person/transmission season after at least 4 rounds of treatment with coverage ≥85 % of eligible population. In these communities 133 children under 5 years of age from Pashopëka (Jénita–Putaco); Hasupiwei (Guaharibos); Arokofita, Kanoshewë, Niayopë and Okiamo (Parima B); Warapawë (Parima C); Masiriki and Toumawei (Parima A), and Kakarama (Shamatari) were negative for Ov-16 (Table 6). This contrasts with the situation in communities with higher *SBR* values (Koyowë and neighbouring villages in Orinoquito and Peñascal, with *SBR* >50,000 bites/person/ transmission season), where 4/85 (5 %) children aged 1–5 years were seropositive in 2013.

Modelling studies conducted with the EPIONCHO transmission model in African savannah settings [43] have suggested that switching to a twice per year treatment strategy during an ongoing annual treatment programme can substantially decrease (nearly by half in highly hyperendemic settings) the additional number of years required to reach the provisional, operational mf prevalence thresholds suggested by the African Programme for Onchocerciasis Control [44] to achieve focal elimination of onchocerciasis. In hyperendemic settings this switch can lead to cost savings. However, these provisional thresholds are not equivalent to transmission breakpoints. A recent comparison of the (stochastic) ONCHOSIM and (deterministic) EPIONCHO models highlights this difference, and indicates that more than 20 years of twice per year treatment (40 treatment rounds) would be required to drive the parasite population to elimination when the initial microfilarial prevalence is greater than 90 % and the coverage of treatment is between 65 and 80 % of the total population (80 to 95 % of eligible individuals) [45]. These projections appear compatible with the results seen in the Amazonian focus in those holoendemic communities in which vector biting rates are very high such as Koyowë. Quarterly treatments may help to accelerate progress to elimination in these communities by further reducing the amount of transmission that takes place between consecutive ivermectin rounds, increasing effective coverage, and exerting a macrofilaricidal effect [19, 20]. Interestingly, the presence of live fertile worms in the host population, as well as of infected (mf positive) people is predicted for 2015 in Koyowë by simulations conducted (by DR) with the EUSIMON model (see [46] for a published precursor), a community with the highest number of treatment rounds (37). This model also predicts the occurrence of transmission and of seropositivity in children by 2015. However, the EUSIMON simulations conclude that the chance of recrudescence in this locality is low if ivermectin treatment were to be interrupted, provided there is no migration of infected people and/or flies into the assumed closed population.

More likely, in the Amazonian focus, and due to the Yanomami network of kinship ties, alliances and hostilities [6, 22], onchocerciasis occurs in a network of interconnected nodes reminiscent of a metapopulation structure, the degree of connection depending both on vector and human movement―the latter being possibly more important and/or better documented. This spatial structure has important repercussions for onchocerciasis transmission and control, as some sub-areas/communities that by themselves may not be able to sustain endemic transmission, may receive an important and periodic influx of heavily infected people from highly endemic areas, making it possible for the infection to persist or be re-introduced. This potential exchange of parasites between otherwise different transmission zones by virtue of the Yanomami micro- and macro-movements [47], may also weaken potential barriers to gene flow. This may allow spread of onchocerciasis from currently non-controlled or less well controlled areas, calling for the development of spatially-explicit, patch transmission models and anthropology-based research avenues for onchocerciasis control in the Amazonian focus. The transport of *Onchocerca* parasites along the reticular nature of the Yanomami use of space may indeed be very diffuse, necessitating intensive treatment in all sub-areas; however, if particular networks could be identified as being responsible for most transmission, a more targeted approach could be beneficial towards the goal of achieving elimination.

### Remaining challenges and directions for future work

The semi-nomadic characteristics of the human population, the remoteness of the Yanomami territory, the holoendemic status of some areas, and the ongoing identification of new endemic communities in the Venezuelan part of the Amazonian focus constitute the main challenges for the elimination of onchocerciasis in the Amazonian focus. From 2009, ivermectin treatment frequency has been increased to four times per year in 80 % of the hyperendemic communities in an attempt to hasten interruption of transmission in areas showing slow progress or in communities recently identified and incorporated into the programme at later stages. Sustaining a high geographical and therapeutic coverage for each treatment round is essential. Only the first round of the 2012–2014 quarterly distributions reached the ≥85 % goal. Besides, since the sentinel and extra-sentinel communities included in this study were partly selected because of relative facility of access, there is the possibility that more remote communities received a lower treatment coverage and/or frequency. Currently, there is a total of 72 communities identified as being remote (~30 % of the total), with a population of 3359 individuals (~47–50 individuals per shapono). This represents approximately 25 % of the total population. Most of these communities are receiving regular treatment, and many of them have received more than 8–12 treatment rounds (Pasumopë, Chalbaud, Mayo and Hashimu sub-areas). However, there are communities located in the Upper Siapa and Upper Ocamo-Parima sub-areas that are only accessible by helicopter (25–30 communities, with ~1000–1500 individuals). This represents only 11 % of the total population and 12 % of all the communities under treatment. Treatment is delivered to these communities when helicopter support is available. Depending on their endemicity status and their connectedness with well-controlled areas, these less accessible communities could pose a risk of infection re-introduction. The use of high spatial-resolution satellite data to identify remote communities in the rainforest is a strategy currently used in an attempt to delineate the extent of the focus, the distribution of transmission zones [6], and the intensification of treatment efforts that will be required to achieve elimination over the entire focus. The prospect of deploying test-and-treat doxycycline treatment as a complementary macrofilaricidal therapy in communities with suppressed transmission has also been considered [48]. Finally, in May 2014, under the auspices of the WHO, Brazil and Venezuela signed a bi-lateral memorandum of understanding calling for a closely coordinated effort between both national programmes in order to reach the goal of onchocerciasis elimination from the Yanomami area. A first joint meeting was held in February 2015, and an action plan for 2015–2016 was agreed and is being implemented [49].

## Conclusions

Our results contribute to the ongoing success of the OEPA strategy [4, 5, 14, 33, 37, 38]. This success has spurred prospects of onchocerciasis elimination in Africa, particularly by increasing coverage and adopting a twice per year treatment strategy [50–54]. However, the OEPA experience may not be fully reproducible in African foci. In the OEPA region treatment has been delivered by mobile teams, who observe directly that ivermectin tablets are ingested by the individuals receiving treatment, circumventing the barriers to elimination posed by the occurrence of systematic non-compliers that affect African countries, particularly those with onchocerciasis-loiasis endemic areas [55]. Currently, no new cases of onchocerciasis-associated blindness have been reported in most of the OEPA region and ocular morbidity has been eliminated from eleven of the 13 previously endemic Latin American foci. Parasite transmission has been interrupted in these eleven foci (~96 % of the total population at risk, representing four of the six countries where the disease was formerly endemic), and elimination has been reached in 10 foci (representing ~78 % of the population at risk). In 2013, onchocerciasis transmission was declared eliminated in Colombia [56], and by 2014 Ecuador became the second Latin American country to attain this goal [4, 33]. In Mexico this target has been reached by 2015 [49, 57] and Guatemala [58] has filed its verification dossier in the WHO. The two countries lagging behind, Venezuela and Brazil, share the most difficult to approach and hard-to-reach populations of the Amazonian focus, yet our results show that given sufficient commitment and determination by the control programme, and unwavering support by OEPA, it is possible to attain and sustain high levels of treatment coverage and increased frequency, attesting to the feasibility of suppressing and ultimately interrupting transmission in the last bastions of onchocerciasis in Latin America.

## Additional file

Additional file 1:
**Detailed description of study area, criteria for elimination, calculation of infection indices and additional entomological data.** **Text S1.** Geographical and environmental characteristics of the Venezuelan part of the Amazonian onchocerciasis focus. **Text S2.** Endemic communities, mapping and geographical information system. **Text S3.** World Health Organization (WHO) criteria for onchocerciasis elimination. **Text S4.** Calculation of parasitological indices. **Text S5.** Calculation of transmission indices. **Table S1.** Number of ivermectin rounds for twice yearly (6-monthly) and quarterly (3-monthly) treatments that reached a therapeutic coverage ≥85 %, by geographical sub-area in the Venezuelan part of the Amazonian focus. **Figure S1.** Monthly biting rates of *Simulium guianense* s.l. recorded in Orinoquito and Parima B during baseline (1982–2000) entomological evaluations. **Figure S2.** Infectivity rates and mean nos. of L3 larvae per fly for *S. guianense* s.l. in Orinoquito and Parima B during baseline (1982–2000) entomological evaluations. **Figure S3.** Monthly infective biting rates and monthly transmission potentials for *S. guianense* s.l. in Orinoquito and Parima B during baseline (1982–2000) entomological evaluations. **Figure S4.** Relationship between the proportion of infective flies and the mean number of infective larvae per fly in *S. guianense* s.l. at baseline. **Table S2.** Comparison of manual dissection and PCR (using PoolScreen™) for estimation of infectivity rates in *S. guianense* s.l., Koyowë, May 2000. (DOCX 129 kb)

## Abbreviations

ABR: annual biting rate
asl: above sea level
ATP: annual transmission potential
AM: arithmetic mean microfilarial load (mf/mg)
APOC: African Programme for Onchocerciasis Control
CAICET: Centro Amazónico de Investigación y Control de Enfermedades Tropicales
CI: confidence interval
CMFL: community microfilarial load (mf/ss)
mf: microfilariae
HBR: hourly biting rate
L3: infective larvae
MFC: prevalence of microfilariae in the cornea
MFAC: prevalence of microfilariae in the anterior chamber of the eye
mg: milligram (of skin)
MBR: monthly biting rate
MDA: mass drug administration
MTP: monthly transmission potential
OEPA: Onchocerciasis Elimination Program for the Americas
PCR: polymerase chain reaction
*R*_0_: basic reproduction ratio (of the parasite)
SBR: seasonal biting rate
s.l.: sensu lato
ss: skin snip
STP: seasonal transmission potential
WHO: World Health Organization
WM: geometric mean (of Williams) microfilarial load (mf/mg)
