# Supplementary material for: Evidence of suppression of onchocerciasis transmission in the Venezuelan Amazonian focus
Source: Parasit Vectors. 2016 Jan 27;9:40. doi: 10.1186/s13071-016-1313-z (PMC4728794; doi:10.1186/s13071-016-1313-z)
Supplement: Additional file 1: — Detailed description of study area, criteria for elimination, calculation of infection indices and additional entomological data. Text S1. Geographical and environmental characteristics of the Venezuelan part of the Amazonian onchocerciasis focus. Text S2. Endemic communities, mapping and geographical information system. Text S3. World Health Organization (WHO) criteria for onchocerciasis elimination. Text S4. Calculation of parasitological indices. Text S5. Calculation of transmission indices. Table S1. Number of ivermectin rounds for twice yearly (6-monthly) and quarterly (3-monthly) treatments that reached a therapeutic coverage ≥85 %, by geographical sub-area in the Venezuelan part of the Amazonian focus. Figure S1. Monthly biting rates of Simulium guianense s.l. recorded in Orinoquito and Parima B during baseline (1982–2000) entomological evaluations. Figure S2. Infectivity rates and mean nos. of L3 larvae per fly for S. guianense s.l. in Orinoquito and Parima B during baseline (1982–2000) entomological evaluations. Figure S3. Monthly infective biting rates and monthly transmission potentials for S. guianense s.l. in Orinoquito and Parima B during baseline (1982–2000) entomological evaluations. Figure S4. Relationship between the proportion of infective flies and the mean number of infective larvae per fly in S. guianense s.l. at baseline. Table S2. Comparison of manual dissection and PCR (using PoolScreen™) for estimation of infectivity rates in S. guianense s.l., Koyowë, May 2000. (DOCX 129 kb) [file 13071_2016_1313_MOESM1_ESM.docx]

**Additional File 1**

**Evidence of suppression of onchocerciasis transmission in the Venezuelan Amazonian focus**

Carlos Botto,^1,2,§^ María-Gloria Basáñez,^3,§,*^ Marisela Escalona,^1^ Néstor J. Villamizar,^1^ Oscar Noya-Alarcón,^1,2^ José Cortez,^1^ Sarai Vivas-Martínez,^4^ Pablo Coronel,^1^ Hortencia Frontado,^5^ Jorge Flores,^6^ Beatriz Graterol,^7^ Oneida Camacho,^1^ Yseliam Tovar,^1^ Daniel Borges,^1^ Alba Lucia Morales,^8^ Dalila Ríos,^8^ Francisco Guerra,^6^ Héctor Margeli,^8^ Mario Alberto Rodriguez,^9^ Thomas R. Unnasch,^10^ and María Eugenia Grillet^11,§,*^

^1^Centro Amazónico de Investigación y Control de Enfermedades Tropicales Servicio Autónomo CAICET, Ministerio del Poder Popular para la Salud, Puerto Ayacucho, Estado Amazonas, Venezuela

^2^Instituto de Medicina Tropical, Facultad de Medicina, Universidad Central de Venezuela, Caracas, Venezuela

^3^London Centre for Neglected Tropical Disease Research, Department of Infectious Disease Epidemiology, Faculty of Medicine (St Mary’s campus), Imperial College London, London, UK

^4^ Cátedra de Salud Pública. Facultad de Medicina (Escuela Luis Razetti), Universidad Central de Venezuela, Caracas, Venezuela

^5^Instituto de Altos Estudios “Dr. Arnoldo Gabaldón”, Ministerio del Poder Popular para la Salud, Maracay, Estado Aragua, Venezuela

^6^Instituto Geográfico de Venezuela “Simón Bolívar”, Caracas, Venezuela

^7^Instituto Nacional de Investigaciones Agrícolas, Puerto Ayacucho, Estado Amazonas, Venezuela

^8^Onchocerciasis Elimination Program for the Americas (OEPA), Guatemala City, Guatemala

^9^Centro de Biotecnología Genómica, Instituto Politécnico Nacional, Reynosa, México

^10^Department of Global Health, University of South Florida, Tampa, Florida, USA

^11^ Laboratorio de Biología de Vectores y Parásitos, Instituto de Zoología y Ecología Tropical, Facultad de Ciencias, Universidad Central de Venezuela, Caracas, Venezuela

^§^Contributed equally to this work

***Corresponding authors:** Laboratorio de Biología de Vectores y Parásitos, Instituto de Zoología y Ecología Tropical, Facultad de Ciencias, Universidad Central de Venezuela, Apartado Postal 47072, Caracas 1041-A, Venezuela. Phone: +58(212)6051404; Fax: +58(212)6051204. *E-mail*: [maria.grillet@ciens.ucv.ve](mailto:maria.grillet@ciens.ucv.ve) (Maria Eugenia Grillet). Department of Infectious Disease Epidemiology, School of Public Health, Faculty of Medicine (St Mary’s campus), Imperial College London, Norfolk Place, London W2 1PG, UK. Phone: +44(207)5943295; Fax: +44(207)4023927. *E-mail*: [m.basanez@imperial.ac.uk](mailto:m.basanez@imperial.ac.uk) (Maria-Gloria Basáñez).

**Detailed description of study area, criteria for elimination, calculation of infection indices and additional entomological data**

**Text S1. Geographical and environmental characteristics of the Venezuelan part of the Amazonian onchocerciasis focus**

“Guayana” means “land of waters”, word derived from an Amerindian linguistic source to depict the rich but complex river system drainage of this biogeographical region [1]. The lowland rainforest river plains and other areas under 500m above sea level (asl) present a climate with a mean annual temperature greater than 24ºC, more than 2,000 mm of mean annual precipitation and only two dry months (December and January) in the year. Hill lands, mountains, and plateau areas above 500m asl show lower temperatures (18–24 ºC) but a similar rainfall pattern. Forest cover accounts for 83% of the total surface area of Venezuelan Guayana and shows an altitudinal gradient: lowland and basimontane forest complex (<400m asl), sub-montane and montane forest complex (400–1200m asl), and savannah with shrubs (800–1200m asl).

**Text S2. Endemic communities, mapping and geographical information system**

More than 250 Yanomami and Sanemá communities were periodically visited during the study in order to record landscape data, proximity to rivers and waterfalls, toponymic information about the community and relevant geographical features such as presence of mountains, nearby rivers, villages (‘shaponos’), kitchen gardens (‘conucos’), and deforested areas.

In addition, the following information was also collected: census data, history of the community (length of time located at the same place; previous names and origins of the community, and history of conflicts, alliances, and patterns of migration). Wherever and whenever possible, data on species composition and biting density of anthropophagic simuliid vectors were also collected.

Geographical coordinates and altitude were recorded with a Garmin GPS in the geographical coordinate system WGS84 datum. Maps of the location of endemic areas and their situation within defined geographical areas and subareas, as well as of sentinel communities (Figure 1 of main text) were prepared in the Lambert Conic Projection using Sirgas-Regven and GRS-80 elipsoide datum. Digital terrain models were obtained from the Shuttle Radar Topography Mission (SRTM, NASA, 2004). River and mountains layers and international borders were provided by the “Instituto Geográfico Venezolano: Simón Bolívar”. Based on geographical features and patterns of settlement and migrations, 12 geographical areas and 31 geographical sub-areas were recognized (Figure 1 and Table 1 of main text).

**Text S3. World Health Organization (WHO) criteria for onchocerciasis elimination**

Four different phases are followed by each country in the Americas to achieve the goal of interrupting transmission and eliminating morbidity with ivermectin mass drug administration (MDA) [2, 3]. *Stage 1* is the phase of *ongoing* transmission, characterized by the presence of *Onchocerca volvulus* infective L3 larvae in vector population samples (fly heads) and evidence of infection in the human population (onchocercal nodules, microfilariae―mf―in skin and eyes and positive serology to parasite antigens, the latter also in children aged <5 years if the force of infection is high). *Stage 2* is characterized by most of the above transmission and parasitological indicators starting to show negative test results, the status of the focus changing to that of *suppressed* transmission. In *Stage 3,* transmission is regarded as *interrupted* when the (overall) focus has reached specific epidemiological indicators such as: i) prevalence of *O. volvulus* mf in the cornea (MFC) and/or anterior chamber (MFAC) of the eye <1%, ii) infectivity rate (presence of L3 larvae in fly heads) by PCR of <1/1,000 (<0.1%) in parous flies or <1/2,000 (<0.05%) in all examined flies (assuming a 50% parous rate), iii) annual transmission potential (ATP) or seasonal transmission potential (STP) <20 L3/person/period, and iv) incidence rate of <1/1,000 individuals (<0.1%) defined as negative serology (measured by Ov-16 antibodies) in school-aged children. In this phase, suspension of treatment is recommended and a 3-year period of post-MDA surveillance is initiated in the focus. *Stage 4* occurs after the 3 years of post-MDA surveillance, when the surveillance parameters have been quantified and all test results confirm that there is no recrudescence. Consequently, onchocerciasis is declared *eliminated* by the WHO after the country requests such certification [2]. This process has taken place in Colombia, Ecuador, and recently Mexico [4, 5, 6].

**Text S4. Calculation of parasitological indices**

**Microfilarial prevalence**

The microfilarial prevalence is reported as the prevalence of skin microfilariae in people aged 5 years and above. We calculated microfilarial prevalence for each community as the crude proportion (not age- and sex-adjusted) of those positive over the number examined expressed as a percent. Binomial 95% confidence intervals (95% CI) for this proportion were calculated by the Clopper-Pearson 'exact' method based on the beta distribution [7]

**Microfilarial intensity**

The arithmetic mean microfilarial load (AM) is the average of the number of microfilariae (including zero counts) *per milligram* (mg) of skin among all those *N* individuals (aged ≥ 5 years) examined in a community at a specific sampling time,

$\text{AM}=\frac{\sum_{i=1}^{N} \left(\frac{m_{i}}{w_{i}}\right)}{N}$, [1]

where is the microfilarial count (sum of the two skin snips taken), and $w_{i}$is the weight of the snips in mg.

The Williams mean (WM) [8] is the geometric mean number of microfilariae (including zero counts) *per milligram* of skin for the whole examined population obtained as follows,

$\text{WM}=\exp\left[\frac{\sum_{i=1}^{N} \ln\left(\frac{m_{i}}{w_{i}}+1\right)}{N}\right]-1$ [2]

The Community Microfilarial Load (CMFL) is the geometric mean number of microfilariae (including zero counts) *per skin snip* in people aged 20 years and above and was the reference index used by the Onchocerciasis Control Programme to assess the intensity of *O.* *volvulus* infection [9],

$\text{CMFL}=\exp\left[\frac{\sum_{i=1}^{n} \ln\left(0.5 m_{i}+1\right)}{n}\right]-1,$ [3]

where we multiply the microfilarial count by 0.5 to obtain the mean per snip and is the number of skin-snipped individuals aged 20 years and older within a community at a specific sampling time.

**Prevalence of microfilariae in cornea and anterior chamber**

The prevalence of microfilariae in the cornea (MFC) and the prevalence of microfilariae in the anterior chamber (MFAC) were calculated as the crude proportions (expressed as percentages) of those positive (for either or both eyes) among those examined. The methods for ophthalmological examination have been described in the main text.

**Seroprevalence of Ov-16**

The prevalence of seropositive children was calculated as the proportion (expressed as a percent) of those with Ov-16 antibodies among those examined and binomial 95% CI were calculated by the Clopper-Pearson method as above [7].

**Text S5. Calculation of transmission indices**

**Hourly biting rate**

The geometric mean number of flies caught per person per hour in each community $j$ $(j=1,...,4)$—a proxy for the hourly biting rate ($HBR_{j}$) to which a person living in the community (Hasupiwei, Pashopëka, Koyowë, Arokofita) would be exposed—was calculated as,

$HBR_{j}=\exp\left\{\sum_{i=1}^{n_{j}} \frac{\ln\left[ (0.5 x_{i,j}/0.833)+1\right]}{n_{j}}\right\}-1$, [4]

where $x_{i}$is the total number of flies caught by the two attractants during each 50-minute collection per hour (multiplied by 0.5 to obtain the mean number of flies landing per person), 0.833 (50/60) is the factor to extrapolate 50 minutes to an hour of collection, and $n_{j}$ is the number of collection hours in community $j$ per transmission season (e.g. 21 for a 3-day collection period; 35 for a 5-day collection period, 84 for a 12-day collection period, etc.).

**Seasonal biting rate**

Following OEPA guidelines [3], the total biting rate for the collection period in each community (referred to as the seasonal biting rate, $SBR_{j}$) was calculated multiplying the geometric mean hourly biting rate per person, $HBR_{j}$, by 10 (the standardised number of potential hours of exposure to blackfly biting during a day) and by the number of calendar days in a transmission season.

**Seasonal transmission potential**

Seasonal transmission potentials for each community $STP_{j}$ were calculated as,

  $SBR_{j}$ $\times$ proportion of flies with *O. volvulus* L3 larvae $\times$ mean no. L3/infective fly.

In an endemic area where multiple rounds of ivermectin treatment have been distributed, and fly infectivity is determined by pool-screening PCR instead of manual dissection, it is assumed that a fly identified as infective carries, on average, one L3 larva in the head.

$STP_{j}$ can be defined as the number of L3 larvae that a person living in community $j$ (Hasupiwei, Pashopëka, Koyowë, Arokofita) would potentially receive if maximally exposed to blackfly bites during the whole transmission season. Values of $STP_{j}$ may be equal to (if transmission is strongly seasonal), or less than, the annual transmission potential ($ATP_{j}$). (Note that if only fly heads are considered for infectivity, the true $STP_{j}$ values may be somewhat higher as L3 larvae present in thoraces and abdomens may be recruited to the site of the bite as the fly takes a blood meal [10].

For the MDA evaluation period we are not able to report $ATP_{j}$ due to *S. guianense* s.l. as entomological collections were not conducted throughout the year. However, it is assumed that parous biting rates and transmission potentials outside of the peak transmission season(s) are substantially lower than those within such season(s), and therefore, the $STP_{j}$ reported are likely to represent fairly accurately (or be proportional to) the $ATP_{j}$ [11].

**Table S1 Number of ivermectin rounds for twice yearly (6-monthly) and quarterly (3-monthly) treatments that reached a therapeutic coverage ≥ 85%, by geographical sub-area in the Venezuelan part of the Amazonian focus.**

| **Geographical sub-area^†^** | | **6-monthly**  **rounds** | **3-monthly**  **rounds** | **Total no. of rounds** |
| --- | --- | --- | --- | --- |
| 13 | Platanal | 21 | 16 | 37 |
| 10 | Manaviche | 20 | 16 | 36 |
| 18 | Orinoquito | 12 | 23 | 35 |
| 21 | Parima A | 9 | 26 | 35 |
| 5 | Middle Ocamo | 21 | 11 | 32 |
| 4 | Lower Ocamo | 17 | 14 | 31 |
| 24 | Shamatari | 10 | 20 | 30 |
| 15 | Guaharibos | 13 | 17 | 30 |
| 19 | Parima B | 8 | 20 | 28 |
| 20 | Parima C | 7 | 21 | 28 |
| 6 | Jénita–Putaco | 11 | 14 | 25 |
| 3 | Ocamo–Orinoco | 22 | 0 | 22 |
| 7 | Upper Ocamo–Shitari | 13 | 9 | 22 |
| 1 | Upper Padamo | 21 | 0 | 21 |
| 25 | Posheno | 1 | 18 | 19 |
| 16 | Peñascal | 5 | 13 | 18 |
| 12 | Mavaquita | 18 | 0 | 18 |
| 11 | Mavaca | 18 | 0 | 18 |
| 9 | Orinoco - Mavaca | 18 | 0 | 18 |
| 14 | Unturán | 5 | 11 | 16 |
| 27 | Chalbaud | 7 | 8 | 15 |
| 23 | Pasumopë | 0 | 10 | 10 |
| 17 | Mayo-Hokotopiwei | 3 | 6 | 9 |
| 22 | Porewë | 0 | 6 | 6 |
| 2 | Upper Cuntinamo | 1 | 5 | 6 |
| 26 | Hashimú | 2 | 1 | 3 |
| 29 | Uasadi | 2 | 0 | 2 |
| 30 | Upper Caura | 0 | 1 | 1 |
| 8 | Upper Ocamo–Parima | 0 | 1 | 1 |
| 28 | Upper Ventuari | 1 | 0 | 1 |
| 31 | Upper Siapa | 0 | 1 | 1 |

^†^The numbering of the geographical sub-areas corresponds to that indicated in Table 1 of the main text.

**Figure S1. Monthly biting rates of *Simulium guianense* s.l. recorded in Orinoquito and Parima B during baseline (1982–2000) entomological evaluations**

The solid green line and squares correspond to the rainforest locality of Koyowë (Orinoquito) and the biting rates for this locality are plotted on the left axis (with a maximum of 35,000 bites per person per month). The dashed red line and solid squares correspond to the highland savannah locality of Niyayowë (Parima B) and the data are plotted on the right axis (maximum 2,000 bites per person per month).

**Figure S2. Infectivity rates and mean nos. of L3 larvae per fly for *S. guianense* s.l. in Orinoquito and Parima B during baseline (1982–2000) entomological evaluations**

The solid red line and circles correspond to the percentage of infective flies (from manually dissected flies carrying L3 larvae in any part of the body) for each month of the year combining data from Koyowë and Niyayowë (plotted on the left axis). The dashed brown line and solid squares correspond to the mean number of L3 larvae per dissected fly (anywhere in the body), plotted on the right axis). The maximum values of L3 prevalence and infection intensity in the fly population correspond to the months of peak transmission.

**Figure S3. Monthly infective biting rates and monthly transmission potentials for *S. guianense* s.l. in Orinoquito and Parima B during baseline (1982–2000) entomological evaluations**

Solid lines and circles represent the monthly infective biting rates (MIBR, on left axis) and dashed lines and solid squares the monthly transmission potentials (MTP, on right axis). Green lines and markers are for the rainforest village of Koyowë; red and brown lines and markers for the highland savannah village of Niyayowë.

**Figure S4. Relationship between the proportion of infective flies and the mean number of infective larvae per fly in *S. guianense* s.l. at baseline**

Observed (solid markers) and predicted (solid line) percentage of infective flies as a function of the mean no. of L3/fly assuming a negative binomial distribution of L3 larvae among flies. Error bars are exact 95% CIs [7]. The degree of overdispersion was assumed to be a linear function of the mean with parameters $k_{0}$= 0.0015 and $k_{1}$= 0.0855, estimated by maximum likelihood. This relationship was derived to provide estimates of infection intensity when only estimates of infection prevalence are available (e.g. those provided by molecular means) as illustrated below.

**Table S1. Comparison of manual dissection and PCR (using PoolScreen^TM^) for estimation of infectivity rates in *S. guianense* s.l., Koyowë, May 2000**

| **Method** | **Number of flies tested** | **Parous rate (%)** | **Number positive** | **Prevalence (%) in total flies (95% CI)** | **Prevalence (%) in parous flies (95% CI)** |
| --- | --- | --- | --- | --- | --- |
| Dissection | 2,729 | 62.8 | 7^a^ | 0.257  (0.103 – 0.528) | 0.408  (0.164 – 0.840) |
| PCR | 3,150 |  | 11^b^ | 0.383  (0.190 – 0.685) | 0.617  (0.306 – 1.103) |

The parous rate of 62.8% was obtained from the fly population sample before splitting it into a fraction for dissection and another for DNA analysis.

^a^number of infective flies; ^b^number of positive pools.

The estimated infectivity rates are in agreement with the baseline infectivity rate obtained for the entire baseline study period, as reported in Table 4 of the main text.

For a prevalence of infection equal to 0.383% as derived from PCR analysis, and according to the lower panel of Figure S4, the mean number of infective larvae per fly would be approximately 0.008 (in the sample dissected manually the recorded mean value was 0.003 L3/fly). For the fly sample analysed by PCR in April 2006, the infectivity rate was 0.071% (Table 4) and the estimated infection intensity would be 0.0009 L3/fly. **References**

1. Huber, O. Geographical and physical features. In: Steyermark JA, Berry PE, Holst BK, editors. Flora of the Venezuelan Guayana, Vol. 1. Missouri Botanical Garden. Oregon: St. Louis & Timber Press, p. 1–61; 1995.
2. World Health Organization. Certification of elimination of human onchocerciasis: criteria and procedures. Geneva: World Health Organization; 2001. Available: <http://whqlibdoc.who.int/hq/2001/WHO_CDS_CPE_CEE_2001.18b.pdf> (accessed 10 November 2015).
3. Program Coordinating Committee and OEPA staff. Guide to detecting a potential recrudescence of onchocerciasis during the post-treatment surveillance period: the American paradigm. Res Rep Trop Med. 2012; 3:21–33. Available: [http://www.oepa.net/Documentos/GuiaVEPT/Guide_Detection_Potential_Recrudescence During_PTS_Englishversion.pdf](http://www.oepa.net/Documentos/GuiaVEPT/Guide_Detection_Potential_Recrudescence%20During_PTS_Englishversion.pdf) (accessed 10 November 2015).
4. World Health Organization. Progress towards eliminating onchocerciasis in the WHO region of the Americas: verification by WHO of elimination of transmission in Colombia. Wkly Epidemiol Rec. 2013; 88:381–385.
5. World Health Organization. Elimination of onchocerciasis in the WHO Region of the Americas: Ecuador’s progress towards verification of elimination. Wkly Epidemiol Rec. 2014; 89:401–408.
6. World Health Organization. Progress towards eliminating onchocerciasis in the WHO region of the Americas: verification by WHO of elimination of transmission in Mexico. Wkly Epidemiol Rec. 2015; 90:577–588.
7. Brown LD, Cat TT, DasGupta, A. Interval estimation for a proportion. Stat Sci. 2001; 16:101–133.
8. Williams CB. The use of logarithms in the interpretation of certain entomological problems. Ann Appl Biol. 1937; 24:404–414.
9. Remme J, Ba O, Dadzie KY, Karam M. A force-of-infection model for onchocerciasis and its applications in the epidemiological evaluation of the Onchocerciasis Control Programme in the Volta River basin area. Bull World Health Organ. 1986; 64:667–681.
10. Renz A. Studies on the dynamics of transmission of onchocerciasis in a Sudan savanna area of North Cameroon. III. Infection rates of the *Simulium* vectors and *Onchocerca volvulus* transmission potentials. Ann Trop Med Parasitol. 1987; 81:239−252.
11. Duke BOL. Studies on factors influencing the transmission of onchocerciasis. IV. The biting cycles, infective biting density and transmission potential of forest *Simulium damnosum*. Ann Trop Med Parasitol. 1968; 62:95−106.
